# Robust Synthesis of Targeting Glyco‐Nanoparticles for Surface Enhanced Resonance Raman Based Image‐Guided Tumor Surgery

**DOI:** 10.1002/smsc.202300154

**Published:** 2024-02-22

**Authors:** Kunli Liu, A. K. M. Atique Ullah, Aniwat Juhong, Chia‐Wei Yang, Cheng‐You Yao, Xiaoyan Li, Harvey L. Bumpers, Zhen Qiu, Xuefei Huang

**Affiliations:** ^1^ Department of Chemistry Michigan State University East Lansing MI 48824 USA; ^2^ Institute for Quantitative Health Science and Engineering Michigan State University East Lansing MI 48824 USA; ^3^ Department of Electrical and Computer Engineering Michigan State University East Lansing MI 48824 USA; ^4^ Department of Civil and Environmental Engineering Michigan State University East Lansing MI 48824 USA; ^5^ Department of Surgery Michigan State University East Lansing MI 48824 USA; ^6^ Department of Biomedical Engineering Michigan State University East Lansing MI 48824 USA

**Keywords:** glyco‐nanoparticles, imaging guided surgeries, surface enhanced Raman spectroscopy, synthesis

## Abstract

Surface enhanced resonance Raman (SERS) is a powerful optical technique, which can help enhance the sensitivity of Raman spectroscopy aided by noble metal nanoparticles (NPs). However, current SERS‐NPs are often suboptimal, which can aggregate under physiological conditions with much reduced SERS enhancement. Herein, a robust one‐pot method has been developed to synthesize SERS‐NPs with more uniform core diameters of 50 nm, which is applicable to both non‐resonant and resonant Raman dyes. The resulting SERS‐NPs are colloidally stable and bright, enabling NP detection with low‐femtomolar sensitivity. An algorithm has been established, which can accurately unmix multiple types of SERS‐NPs enabling potential multiplex detection. Furthermore, a new liposome‐based approach has been developed to install a targeting carbohydrate ligand, i.e., hyaluronan, onto the SERS‐NPs bestowing significantly enhanced binding affinity to its biological receptor CD44 overexpressed on tumor cell surface. The liposomal hyaluronan (HA)‐SERS‐NPs enabled visualization of spontaneously developed breast cancer in mice in real time guiding complete surgical removal of the tumor, highlighting the translational potential of these new glyco‐SERS‐NPs.

## Graphical abstract

A method is reported to produce colloidally stable and bright surface‐enhanced resonance Raman nanoparticles (SERS‐NPs). Furthermore, a new liposome‐based approach has been developed to install a targeting carbohydrate ligand, that is, hyaluronan (HA), onto the SERS‐NPs bestowing significantly enhanced binding affinity to its biological receptor CD44 overexpressed on tumor cell surface, enabling image‐guided surgery to completely remove breast cancer.

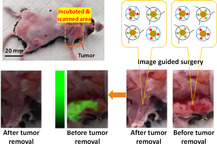

## 1 Introduction

Raman spectroscopy is an attractive optical technique to simultaneously detect multiple targets of interest. However, the low inherent sensitivity of Raman hinders its wide application. To overcome this drawback, Raman active dyes can be deposited onto noble metal surface.^[^ ^1^, ^2^ ^]^ An up to 10 orders of magnitude enhancement of Raman signals can be potentially observed, which is referred to as surface enhanced resonance Raman (SERS).^[^ ^1^, ^2^, ^3^ ^]^ Au‐nanoparticles (Au‐NPs) coated with Raman dyes have been one of the most popular types of SERS‐NPs investigated,^[^ ^4^ ^]^ which have been applied in many areas, including chemical analysis, environmental monitoring, and medical diagnostics.^[^ ^5^ ^]^

To prepare Au‐based SERS‐NPs, the Au‐NP cores have been most commonly synthesized *via* the sodium citrate reduction method,^[^ ^6^ ^]^ which was followed by the absorption of Raman active probes onto the NP surface.^[^ ^4^, ^7^, ^8^ ^]^ As the core sizes of Au‐NPs are known to significantly impact Raman signal enhancement,^[^ ^9^, ^10^ ^]^ it would be ideal that the SERS‐NPs synthesized have narrow size distributions. Furthermore, the NPs should be stable in biological media, which tend to foul the surface of the particles resulting in NP aggregation and significantly reduced SERS properties. Methods that can generate bright and colloidally stable SERS‐NPs with uniform size and shape distribution are important.^[^ ^1^, ^2^, ^4^, ^11^, ^12^ ^]^

For biological detections aided by SERS‐NPs, another important parameter is the selective binding of NPs to the target of interests.^[^ ^13^ ^]^ In order to accomplish this, ligands such as monoclonal antibodies can be conjugated to NP surface to aid in targeting.^[^ ^14^, ^15^ ^]^ Several strategies have been developed to install targeting ligands including through the high affinity interactions of gold and sulfhydryl group^[^ ^16^ ^]^ or coating of the NP with a layer of silica to introduce reactive functional groups onto the NP surface.^[^ ^17^ ^]^ For example, recently, the impressive synthesis of a library of 26 gold‐based SERS‐NPs has been reported, which aided in the detection of multiple types of tumor cells.^[^ ^17^ ^]^ In contrast, with the high cost and the relative ease of denaturing of monoclonal antibodies, other types of targeting ligands can be explored.

Herein, we report a synthetic strategy to prepare bright and colloidally stable SERS‐NPs, which is applicable to a wide range of Raman dyes (flavors). An algorithm has been adapted to decode the resulting library of SERS‐NPs with multiple flavors through their respective characteristic Raman fingerprints. Furthermore, as an alternative to monoclonal antibodies, a readily available carbohydrate, i.e., hyaluronan (HA) has been investigated as the targeting ligand on SERS‐NPs. However, the direct attachment of HA onto SERS‐NPs failed to produce significant targeting of the biological receptor, CD44 protein, overexpressed on tumor cells. To overcome this obstacle, a new liposomal based synthesis approach has been established that led to a new type of HA‐SERS‐NP with significantly enhanced CD44 binding. The liposomal HA‐SERS‐NP can effectively guide the complete surgical removal of breast cancer spontaneously developed in mice in real time through Raman imaging, highlighting its translational potential.

## 2 Results and Discussion

### 2.1 Development of a Robust Synthesis Protocol for SERS‐NPs

Our synthesis of SERS‐NPs started from the formation of Au‐NP cores followed by coating with a Raman active dye. The first synthetic approach we tested utilized the common method of sodium citrate reduction of tetrachloroauric(III) acid to prepare NPs with an average of 50 nm diameter.^[^ ^18^, ^19^ ^]^ The Au‐NPs formed were then incubated with a non‐resonant Raman dye such as S420.^[^ ^18^ ^]^ However, these NPs gave little SERS signals at pM NP concentration, which was presumably because of the low amounts of dye attached to the Au‐NPs with this synthetic procedure in our hands. To improve the SERS intensities, a variety of synthetic conditions were examined,^[^ ^20^ ^]^ which included varying the timing of dye addition *versus* the formation of Au‐NPs, and the addition of additives such as ammonia hydroxide to stabilize Au‐NPs formed before the installation of the Raman dye. However, none of these efforts yielded stable NPs with strong SERS signals.

Another complication observed in the synthesis was the inhomogeneity of the NP core formed. It is known that the optimal core diameters of the Au‐NPs around 50 nm are ideal for maximum SERS enhancement.^[^ ^10^ ^]^ When the Au‐NPs synthesized were larger than 30 nm through the sodium citrate reduction method, significant heterogeneities in shape and size of Au‐NPs were observed (Figure S2, Supporting Information), which were consistent with literature reports.^[^ ^21^, ^22^ ^]^

**Figure 1:**
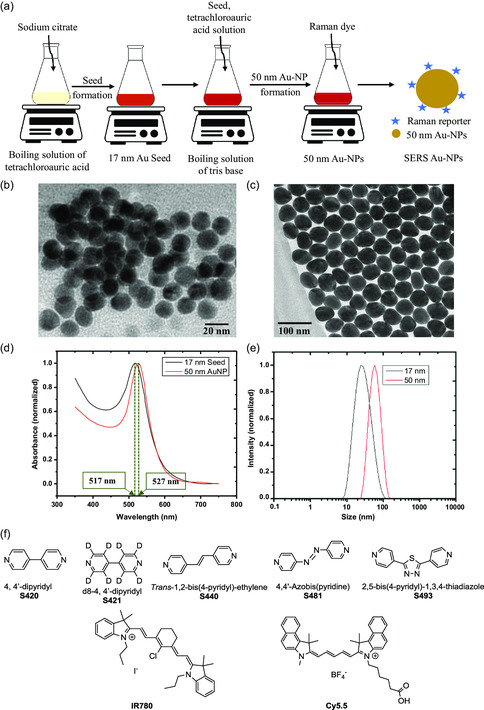
Synthesis and characterizations of SERS‐NPs. a) Schematic illustration of the tris base assisted synthesis of SERS‐NPs. Au‐NP seeds were first produced *via* the sodium citrate reduction method, which was followed by seed mediated growth *via* tris base leading to 50 nm Au‐NPs at 100 °C. The Raman dye was added right after the formation of 50 nm Au‐NPs and stirred for 1 min, followed by immediate cooling down in an ice bath. This method was applicable for both non‐resonant and resonant dyes yielding bright SERS signals with the concentrations of dyes needed differing significantly between non‐resonant dyes (10 μM) and resonant dyes (50 nM). b) TEM images of Au‐NP seeds and c) SERS‐NPs. d) UV‐Vis spectra and e) hydrodynamic diameters of Au‐NP seeds and SERS‐NPs in MilliQ water at room temperature. f) Structures of non‐resonant dyes (S420, S421, S440, S481, and S493) and resonant dyes (IR780, and Cy5.5) examined for SERS‐NP formation.

The aforementioned difficulties encountered in SERS‐NP synthesis prompted us to examine alternative procedures. While there are many methods for the preparation of Au‐NPs,^[^ ^21^, ^23^, ^24^, ^25^, ^26^, ^27^, ^28^, ^29^, ^30^ ^]^ only a few of them have been applied to the formation of SERS‐NPs with strong SERS signals applicable to multiple Raman dyes. After exploring various synthesis strategies, the seed mediated growth method with tris base^[^ ^31^ ^]^ (**Figure** **1**) turned out to be the best in our hands.

For the seed‐mediated growth strategy, Au‐NP seeds with average diameters of 17 nm were synthesized first with sodium citrate as the reducing agent. The Au‐NP seeds were then mixed with a solution of tris base heated at 100 °C followed by the addition of tetrachloroauric acid, which triggered NP growth to form 50 nm Au‐NPs by tuning the number of seeds versus the amount of tetrachloroauric acid added. To install the Raman dye, we discovered that upon formation of the 50 nm Au‐NPs, a dye such as S420 (10 μM) should be immediately added to the NP solution at 100 °C. The resulting SERS‐NPs were stable in solution and gave bright Raman signals. The schematic illustration of the synthesis of SERS‐NPs was shown in Figure 1a. Addition of the dye after the Au‐NP solution had cooled down, before or during seeded growth of the NPs, failed to produce SERS‐NPs with strong Raman signals. Thus, the small‐time window for Raman dye installation right after the formation of Au‐NPs is critical, presumably because the nascent Au‐NP surface has a significant number of vacant sites for the Raman dye to bind. Au‐NP seeds and SERS‐NPs were characterized by UV‐Vis spectroscopy, transmission electron microscopy (TEM), dynamic light scattering (DLS), and ζ potential analyses (Figure 1b–e and Table S1, Supporting Information). These SERS‐NPs were more homogeneous in size distribution with average diameters of 50 nm compared to those prepared directly *via* the citrate reduction method (Figure 1c and Figure S2, Supporting Information). The surface plasmon resonance bands observed at 517 and 527 nm were also an indication of the formation of 17 nm Au‐NP seeds and 50 nm SERS‐NPs respectively (Figure 1d).^[^ ^32^ ^]^ The size distribution of the 50 nm SERS‐NPs prepared *via* the seed mediated synthesis was further validated from their hydrodynamic size measured by dynamic light scattering analysis (Figure 1e). The ζ potentials of the SERS‐NPs were around −35 mV, which was more positive than that of the NP seed (−41 mV) presumably due to the displacement of negatively charged sodium citrate from NP surface by tris base (Table S1, Supporting Information).^[^ ^31^ ^]^

**Figure 2:**
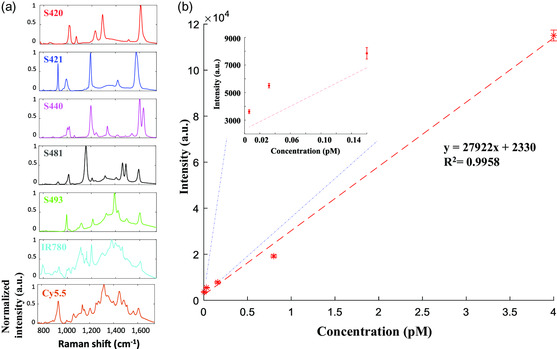
a) Raman spectra of SERS‐NPs (S420, S421, S440, S481, S493, IR780, and Cy5.5). b) The intensities of the highest peaks of Raman spectra of SERS (S440)‐NP correlated linearly with NP concentrations. The insert in the figure shows that SERS (S440)‐NP can be sensitively detected at low fM concentrations.

The generality of our synthesis procedure for both non‐resonant and resonant Raman dyes was investigated. SERS‐NPs bearing non‐resonant Raman dyes^[^ ^33^ ^]^ S420, S421, S440, S481, and S493 (Figure 1f and S1, Supporting Information) respectively showed strong SERS Raman signals. Despite the similarity in structures of some Raman dyes (e.g., S420 vs. S421), these dyes have distinct fingerprint patterns in their respective Raman spectra potentially enabling multiplexing (**Figure** **2**a). For resonant Raman dyes (dyes with absorbance overlapping with the wavelength of the incident light) such as IR780, interestingly, when 10 μM of the IR780 dye was used as for the non‐resonant dye, it led to instantaneous aggregation of Au‐NPs. Upon careful optimization, it was discovered that 50 nM of IR780 was sufficient to produce bright NPs. This supported the idea that with the extended conjugated structure, a resonant Raman dye can have a higher affinity to Au‐NPs as compared to a non‐resonant Raman dye. Besides IR780, resonant Raman dyes IR792 and Cy5.5 also produced NPs with strong signals with IR792 giving similar SERS spectrum as that of IR780.

### 2.2 Detection Limit Analysis and Ratiometric Quantification of Mixtures of Various Flavors of SERS‐NPs

Detection limits of the SERS‐NPs synthesized were evaluated. The intensities of Raman signals from solutions (200 pM, 100 pM, 20 pM, 4 pM, 800 fM, 160 fM, 32 fM, and 6 fM) containing various flavors of SERS‐NPs (S420, S421, S440, S481, S493, IR780, and Cy5.5 (Figure 1f)) were measured. These Raman dyes were selected due to their distinct Raman signals and commercial availabilities. As shown in Figure 2b, even at 6 fM concentration, NP such as S440 SERS‐NP could still be detected.

An important application of the SERS‐NPs is to use a library of SERS‐NPs to detect multiple targets simultaneously. In order to accomplish this, an unmixing algorithm needs to be established. Phantoms with mixtures of three flavors of SERS‐NP at seven different ratios of the SERS‐NPs were prepared (Figure S3a, Supporting Information). Three sets of spectra were acquired: 1) spectra of the solutions of various mixed NPs; 2) the reference spectrum for each flavor; and 3) background spectra, i.e., the spectra of the plastic tube container filled with water. Codes were prepared and applied to demultiplex the components, which accurately determined the ratios of the various flavors of the particles in the mixture (Figure S3b, Supporting Information).

### 2.3 Ligand Attachment to SERS‐NPs

For biological detection, it is desirable that targeting ligands can be attached to the SERS‐NPs. The most common ligand type investigated for SERS‐NPs to date is monoclonal antibodies.^[^ ^17^ ^]^ While antibodies can bestow high specificity in target binding, they can be expensive especially for pre‐clinical studies (hundreds of $ per 100 μg) and can denature upon extended storage. Although the cost of monoclonal antibodies can be potentially managed with wide clinical adaption, as an alternative, we explored polysaccharides such as HA^[^ ^34^ ^]^ as the targeting ligand. HA is readily available commercially (≈$200/g), can be stored for extended periods, and is biocompatible as highlighted by its common usage in the cosmetic industry. A major receptor of HA in human bodies is the glycoprotein CD44.^[^ ^35^ ^]^ On normal cells, CD44 exists primarily in the inactive low affinity form exhibiting minimal binding with HA,^[^ ^36^ ^]^ which can be converted to the active high HA affinity structure in the presence of inflammatory signals.^[^ ^37^ ^]^ In contrast, tumor‐derived cells often express CD44 in its high‐affinity state capable of constitutively binding HA,^[^ ^38^ ^]^ and the high affinity CD44 is found over‐expressed on the surfaces of a wide range of cancer cells.^[^ ^39^, ^40^, ^41^, ^42^ ^]^ HA has been conjugated to a variety of NPs for imaging applications such as magnetic resonance imaging and fluorescence^[^ ^43^, ^44^, ^45^, ^46^ ^]^ or in vitro enzyme detection.^[^ ^47^ ^]^ However, to the best of our knowledge, it has not been utilized for in vivo SERS‐NP based detection.

To attach HA on SERS‐NPs, the first approach attempted was to coat SERS‐NPs with a layer of silica to introduce functional groups such as amine onto NPs.^[^ ^18^ ^]^ The Stöber method is one of the most common approaches for silica coating on NPs through the hydrolysis of tetraethoxysilane (TEOS) with ammonia hydroxide as the base.^[^ ^48^ ^]^ However, in our synthesis, the addition of ammonia hydroxide to promote the silica formation on NPs significantly reduced SERS signals of the resulting NPs presumably due to desorption of Raman dye from NPs in the process. Increasing the concentration of the Raman dye to 1 mM during TEOS coating of SERS‐NPs did not improve the Raman signals. While silica‐coated SERS‐NPs have been reported,^[^ ^17^, ^49^ ^]^ our results are consistent with the findings that silica coating can significantly reduce SERS intensities.^[^ ^9^, ^50^ ^]^

As an alternative to silica coating to attach biomolecules to Au‐NP surface, ligands can be functionalized with sulfhydryl groups by taking advantage of the strong gold‐thiol interactions.^[^ ^51^, ^52^, ^53^, ^54^, ^55^ ^]^ HA (average MW: 10 kDa) was thiolated and incubated with the SERS‐NPs overnight followed by repeated centrifugation to remove unbounded HA.

Colloidal stability of NPs is important for biological applications. While the uncoated SERS‐NPs were readily dispersed in water, these particles aggregated in PBS buffer as evident from the precipitation observed and the loss of color in the supernatant (Figures S4ai vs. S4aii, Supporting Information). HA coating of SERS‐NPs significantly improved the colloidal stability of the particles with HA‐SERS‐NPs stable in PBS buffer with no precipitation for more than 6 months (Figure S4aiii, Supporting Information). Consistent with HA installation, there was an increase in the hydrodynamic diameter and change in the ζ potential of the HA‐SERS‐NPs as compared to the uncoated SERS‐NPs (Figure S4b and Table S1, Supporting Information). To confirm that HA immobilized retained their biological binding, a competitive enzyme linked immune‐sorbent assay (ELISA) was set up,^[^ ^56^, ^57^ ^]^ where the HA‐SERS‐NPs were used to compete against native HA polysaccharide for binding with immobilized CD44 in ELISA wells (Figure S4c,d, Supporting Information). As SERS‐NPs are often applied at relatively low concentrations (pM), the HA‐SERS‐NPs formed with this direct coating method were assayed up to 250 pM concentrations. However, these HA‐SERS‐NPs showed relatively weak affinities for CD44 as evident from the significant (≈50%) CD44 binding remaining even with 250 pM of SERS‐NPs (Figure S4d, Supporting Information). To test the possibility that Raman dye installed on the SERS‐NPs interfered with HA binding, thiolated HA was also incubated with Au‐NPs without Raman dye. The resulting NPs did not have stronger binding with CD44 in the competitive ELISA assay either (Figure S4e, Supporting Information). The usage of higher molecular weight HA (MW: 250 and 1500 kDa) for NP coating did not lead to improvement in binding (Figure S4e, Supporting Information). The weak avidity of such HA‐NPs with CD44 was presumably due to the insufficient amounts of HA immobilized on NP surface.

**Figure 3:**
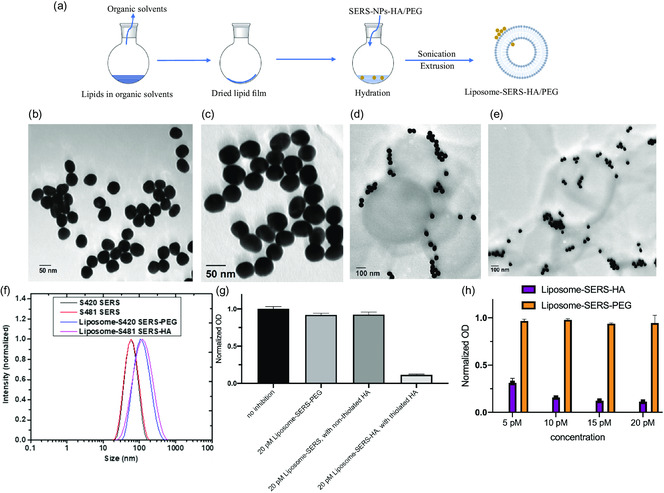
Synthesis and characterizations of liposome‐SERS‐HA and liposome‐SERS‐PEG. a) Schematic illustration of the synthesis of liposome‐SERS‐HA/PEG. b) TEM images of S420 SERS‐NP, c) S481 SERS‐NP, d) liposome‐S420 SERS‐PEG, and e) liposome‐S481 SERS‐HA. SERS‐HA/PEG NPs were anchored on the surface of the liposome. f) DLS data of SERS‐NPs and liposome‐SERS‐HA/PEG. The increased size is an indication of liposome anchoring. g) Competitive ELISA showed that HA attachment was successful only with thiolated HA. Strong competition was observed at 20 pM liposome‐SERS‐HA. h) Liposome‐SERS‐HA could compete against HA for CD44 binding in a dose dependent manner from 5 to 20 pM, while the corresponding liposome‐SERS‐PEG showed no effects at the equivalent concentrations. The error bars represent the standard deviations from three experiments.

We explored various methods to enhance the affinity of SERS‐HA‐NPs with CD44. We envision that stronger binding may be achieved with higher loading of HA on the NPs. To accomplish this, we explored liposomal formulation^[^ ^58^ ^]^ of SERS‐NPs, which have not been investigated before. The thin‐film hydration method was applied first using a solution of SERS‐NPs to hydrate the lipid film. However, extensive NP aggregation was observed during the hydration process, as indicated by the color change to black. To overcome this, SERS‐NPs were incubated with thiolated HA (HS‐HA) first, which were then used to hydrate the lipid film (**Figure** **3**a). No significant absorbance change or aggregation (*via* UV‐Vis absorption and hydrodynamic diameter) was observed for liposome‐SERS‐HA after 24 h of incubation (Figure S5, Supporting Information) in PBS and serum. Thus, the resulting liposome‐SERS‐HA were colloidally stable in PBS buffer and serum. As a control, thiolated polyethylene glycol (PEG‐SH, MW 5 kDa) was immobilized onto SERS‐NPs following the same liposomal formulation strategy as liposome‐SERS‐HA (Figure 3a, Supporting Information). The TEM images of the SERS‐NPs before and after the liposomal complex formation showed that SERS‐NPs were bound on the surface of liposome (Figure 3b–e). The formation of liposomal complexes was evidenced from the increase of the hydrodynamic diameter (Figure 3f) and change in ζ potential (Table S1, Supporting Information). Interestingly, this new liposome‐SERS‐HA exhibited strong CD44 binding in the competitive ELISA assay with the avidity of CD44 significantly improved compared to the HA‐SERS‐NPs formed without liposome formulation as 15 pM of liposome‐SERS‐HA almost completely inhibited HA binding with CD44 with a dose dependent response (Figure 3g,h vs. Figure S4d, Supporting Information). HA thiolation was important as liposome‐SERS‐NPs obtained with the non‐thiolated HA did not compete against HA for CD44 binding (Figure 3g). It is known that HA can interact with phospholipids through hydrogen bonding and hydrophobic interactions with HA distributed in punctate patterns on lipid bilayer surface.^[^ ^59^, ^60^ ^]^ The sulfhydryl groups in thiolated HA may cluster HA around SERS‐NPs on the liposomes enhancing the avidity with CD44. Under the same experimental conditions, liposome‐SERS‐PEG exhibited no competition against HA in the competitive ELISA assay either, highlighting the important role of HA for SERS‐HA binding with CD44 (Figure 3h).

The amount of HA on the liposome‐SERS‐HA was quantified. Comparison of the CD44 binding by liposome‐SERS‐HA versus HA polymer showed that 20 pM of liposome‐SERS‐HA contained an equivalent of 2.2 μM HA, which corresponded to 18% w/w. In parallel, the amount of HA attached to HA‐SERS‐NPs was quantified by the carbazole assay following acid cleavage of HA from the NPs,^[^ ^61^ ^]^ which gave the average HA loading of 5%. Higher loading percentage of HA determined from the competitive ELISA assay may be due to the enhanced multi‐valency effect after HA attachment to the SERS‐NPs, which may be through thiol‐gold interaction^[^ ^51^ ^]^ and HA binding to liposome.^[^ ^62^ ^]^

### 2.4 Stability and Biocompatibility of SERS‐NPs in Biological Milieu

For biological applications, it is important that the NPs are stable and biocompatible in biological milieu. In vitro cytotoxicity assay was performed to evaluate the toxicity of SERS‐NPs against both 4T1 mouse breast cancer cells and HC11 normal mouse mammary cells. The 3‐(4,5‐Dimethylthiazol‐2‐yl)‐5‐(3‐carboxymethoxyphenyl)‐2‐(4‐sulfophenyl)‐2H‐tetrazolium (MTS) cell viability assay showed that SERS‐NPs were not toxic up to the highest concentration tested (500 pM) (Figure S6, Supporting Information). In addition, the colloidal stabilities of the SERS‐NPs were examined under various conditions. As shown in Figure S5 (Supporting Information), UV‐Vis spectroscopy and DLS measurements of HA‐SERS‐NP showed little changes in NP sizes under high salt (10 × PBS) and 50% serum incubation suggesting the high stability of the particles. There were no significant changes in SERS intensities either when stored at 4 °C over 3 weeks (Figure S5c, Supporting Information).

### 2.5 Targeted SERS Imaging of Breast Cancer Cells

**Figure 4:**
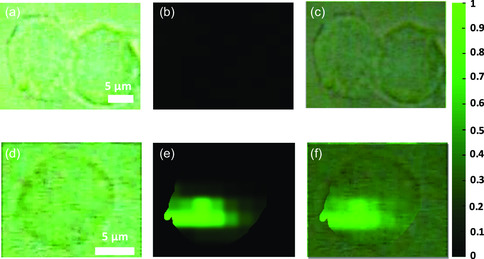
Images of 4T1 cells incubated with liposome‐S440 SERS‐PEG (panels a–c, control without targeting) and liposome‐S421 SERS‐HA (panels d–f. HA is used for targeting CD44 protein overexpressed in 4T1 cells) upon removal of unbound particles. a) Bright‐field microscopy image of 4T1 cells, b) S440 SERS image of 4T1 cells, c) overlay of a and b images. d) Bright‐field microscopy image of 4T1 cells, e) S421 SERS image of 4T1 cells, f) overlay of d and e images. The spectra were recorded using 50X objective lens with 10 × 10 number of pixels, 5 frames accumulation, 2 μm step size, and 1 s exposure time. Colorimetric weight factors in panel b indicated that there were no SERS signals obtained demonstrating no non‐specific binding of liposome‐S440 SERS‐PEG by the cells was detected. In contrast, the significant signals from panel e suggest the significant binding of liposome‐S421 SERS‐HA with 4T1 cells.

The in vitro SERS imaging of breast cancer cells was carried out using liposome‐SERS‐HA with liposome‐SERS‐PEG as the control. Breast cancer cell 4T1 was explored as the target cell, which expresses a high level of CD44 on the surface.^[^ ^63^, ^64^ ^]^ 4T1 cells were incubated with solutions of liposome‐SERS‐PEG and liposome‐SERS‐HA respectively under the same experimental conditions. The cells were then rinsed with the buffer to remove the unbound particles, and Raman signals were recorded. Cells incubated with liposome‐S440 SERS‐PEG did not show any significant Raman signals (**Figure** **4**a–c) indicating little non‐specific binding of liposome‐SERS‐PEG with the cells. In contrast, strong Raman signals were obtained from the cells incubated with the liposome‐S421 SERS‐HA (Figure 4d–f) demonstrating the binding of liposome‐SERS‐HA to the breast cancer cells. Similarly, other flavors of SERS‐NP could also be used for the HA dependent detection of 4T1 cells demonstrating the generality (Figure S7 and S8, Supporting Information).

### 2.6 Liposome‐SERS‐HA Enabled Successful Image‐Guided Surgery of Breast Cancer Spontaneously Developed in Mice

Breast cancer is the leading cause of cancer and the second most frequent cause of death in female cancer patients. Breast conserving surgery (BCS), also referred to as lumpectomy, is the most widely used surgical procedure and standard of care for a majority of breast cancer.^[^ ^65^ ^]^ BCS aims to completely remove the tumor while preserving surrounding healthy breast tissue. However, cancer often grows in an irregular shape, and there may be microscopic components extending from the main tumor, rendering it challenging to ensure complete tumor resection. Breast surgical oncologists typically rely on palpation and experience during surgery to determine the tumor margin. Intraoperative frozen section assessment can be performed,^[^ ^66^ ^]^ but it can significantly increase the total time needed for the operation as it is time consuming for the pathologist to evaluate the ill‐defined cancer. Furthermore, some cancers are so small or nonpalpable that frozen section is not performed for fear of losing tumor specimen. After surgery, the tissues removed are commonly subject to histological analysis. If tumor is observed at the margin (termed “positive margin”), it would indicate that there is residue tumor left at the surgical sites and there are increased risks of in‐breast tumor recurrence, thus necessitating additional surgery.^[^ ^67^ ^]^ Re‐excision requires a second surgical trip, additional stress to the patient, and sometime weeks before the patient can be determined to have had a cancer‐free resection. The re‐excision lumpectomy rate for some individual surgeons within the United States can be as high as 70%.^[^ ^68^ ^]^ Thus, a method that can image tumor intraoperatively and aid the surgeon to completely remove tumor without significantly increasing in‐surgery time will be tremendously beneficial.

SERS‐NPs have been investigated to image freshly excised mouse breast cancer tissues ex vivo to provide feedback for tumor removal.^[^ ^69^, ^70^ ^]^ It would be more desirable that the surgical sites can be directly imaged during surgery in real time. In addition, current studies on SERS‐NPs guided surgery have only utilized xenografted mouse breast cancer model rather than the spontaneous breast cancer.^[^ ^69^, ^70^ ^]^

For our study, to more closely mimic human breast cancer clinical conditions, we obtained the mouse mammary tumor virus promoter (MMTV)‐polyoma virus middle T (PyMT) mice. These mice express the PyMT antigen with the MMTV promoter/enhancer.^[^ ^71^, ^72^ ^]^ As a result, all female PyMT/MMTV mice spontaneously develop multiple palpable mammary tumors within 4–6 months with a more native breast cancer microenvironment and tumor morphology as compared to xenografted breast cancer.

With the tumor bearing mice, breast conserving surgery was performed by first removing the tumor surgically guided by palpating the tumor tissue area. A solution of liposome‐S421 SERS‐HA was then sprayed on the tumor site of resection to detect residual tumor. After 15 min, the surgical site was rinsed with buffer to remove unbound particles. Raman signals were observed in the tumor site. However, it was unclear to what extent the non‐specific binding of NPs by the tissues played in the retention of the NPs in the surgical site.

**Figure 5:**
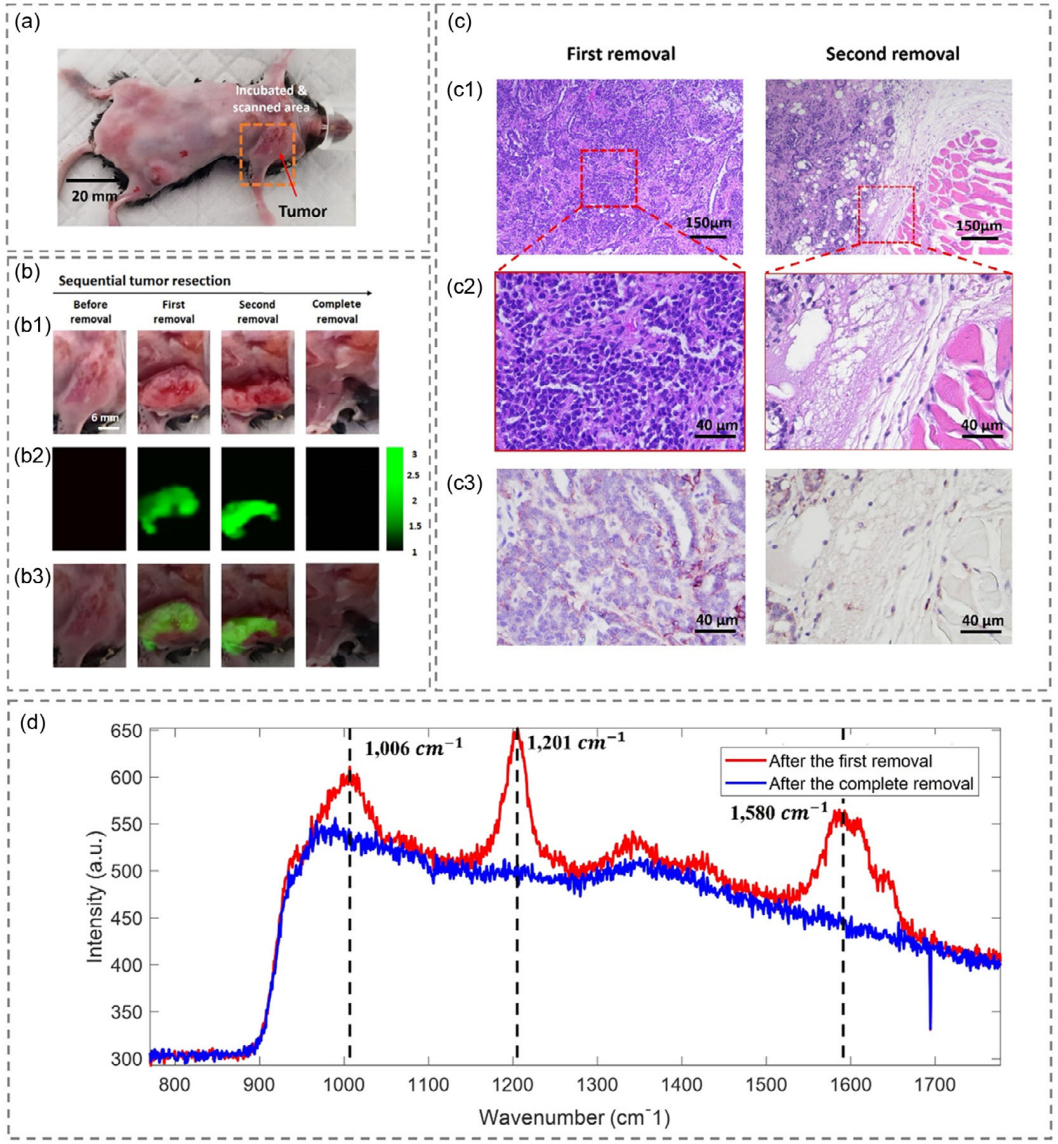
Raman image‐guided surgery using the mixture solution of liposome‐S421 SERS‐HA and liposome‐S440 SERS‐PEG. a) Photograph of the tumor bearing mouse before surgery; b) Sequential tumor resection, b1) photographs of the tumor area, b2) the ratiometric Raman (S421/S440) images, b3) overlaid ratiometric images with photographs the tumor areas before surgery and after each of the three surgical removals of the tumor; The scale bar is for S421/S440 weight ratio. c) H&E and IHC images of resected tumors from (b), c1–c2) H&E images of the tumor acquired by 10X and 40X magnification, respectively, and c3) the corresponding IHC images of CD44 in the tumor acquired by 40X magnification; d) representative Raman spectra of the tumor area after the first tumor removal and after the complete tumor removal (the vertical black dashed lines mark the characteristic SERS peaks of S421 with the wavenumbers given).

To reduce the influence of non‐specific binding and more accurately depict tumor in mice, we mixed the liposome‐S440 SERS‐PEG with liposome‐S421 SERS‐HA at 1:1 ratio. Tumor bearing MMTV‐PyMT mice were subjected to tumor removing surgery, which was followed by spraying of the mixed NP solution and removal of unbound particles through washing. After the first surgical removal of the tumor, particle spraying and washing off unbound particles, S440 signals were observed not only in part of the tumor site but also in some of the surrounding normal tissues presumably reflecting the non‐specific retention of the NPs by tissues. To more precisely define the tumor location, the Raman signals from the surgical site were unmixed to calculate the ratio of the signal intensities from S421 over S440. Hotspots with significantly higher S421 signals over those of S440 in ratiometric images of the tumor would be indicative of tumor presence attributed to HA binding with CD44 expressed in tumor. After surgical removal of tumors three times, the surgical site was free of tumor signals (**Figure** **5**). The tumor removed and corresponding IHC and H&E images of resected tumor were shown in Figure 5.

**Figure 6:**
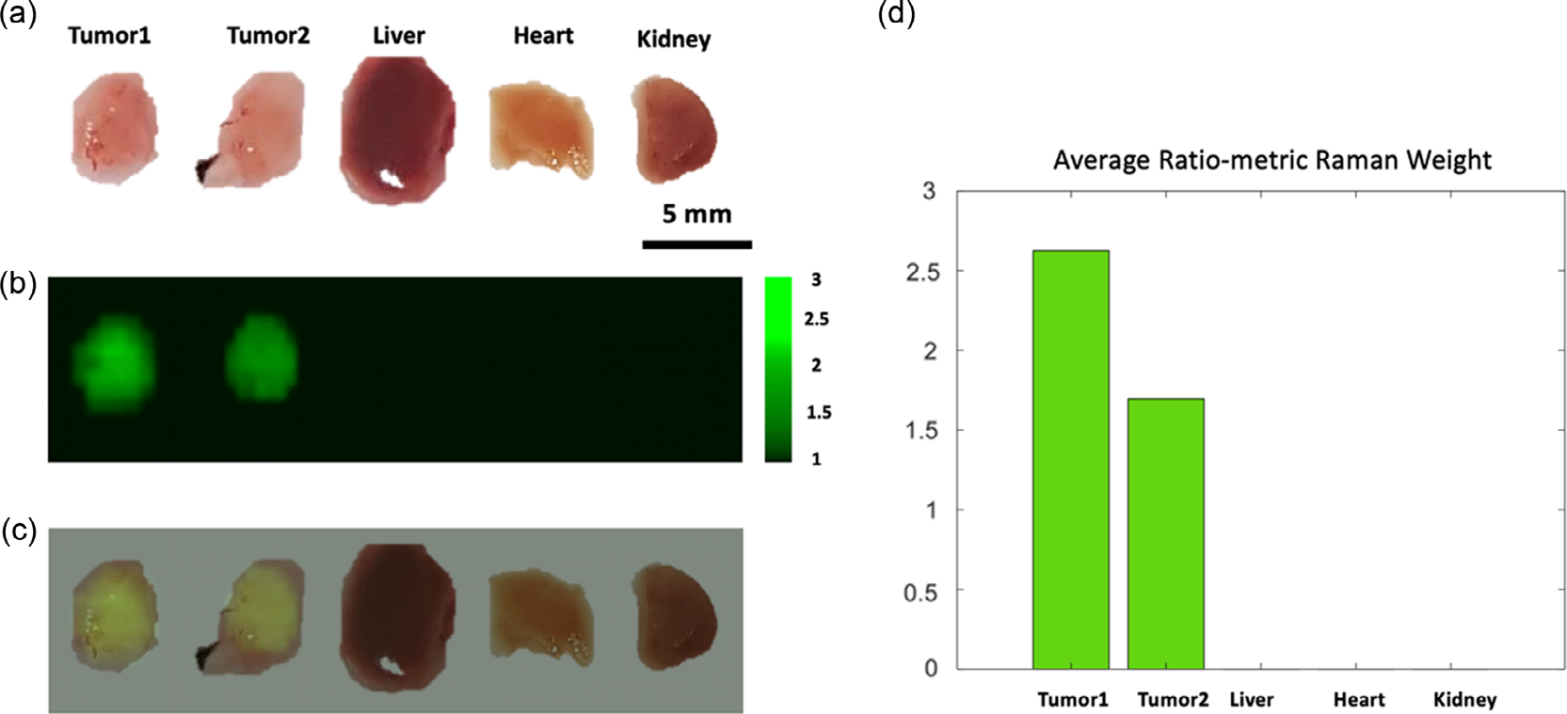
Ratiometric images of the mixture solution of liposome‐S421 SERS‐HA and liposome‐S440 SERS‐PEG applied on the tumor and control tissues. a) Photograph of the tissues; b) Ratiometric Raman images of signal intensities of S421 over those of S440; The scale bar is for S421/S440 weight ratio. c) Overlaid images of the photograph and the ratiometric Raman images; d) The average values of ratiometric Raman weight from various tissues. The much higher intensities from the tumor sites confirm the abilities of the liposome‐S421 SERS‐HA to target tumor.

To confirm the in vivo imaging results in mice, ex vivo staining was carried out with the surgically removed tumor, and other tissues including liver, heart, and kidney. Resected tissues were stained with a mixture of liposome‐S421 SERS‐HA and liposome‐S440 SERS‐PEG followed by repeated washing with PBS buffer to remove unbound particles. Raman imaging was performed and the ratiometric images were generated (**Figure** **6**). Preferential accumulation of liposome‐SERS‐HA over liposome‐SERS‐PEG was observed on tumor tissue, but not in liver, kidney or heart tissue. The results from ex vivo staining corroborated with the in vivo images and the targeting ability of liposome‐SERS‐HA particles.

There are limitations to our study. While many breast cancer cells express CD44,^[^ ^39^, ^73^ ^]^ due to tumor heterogeneity, it is possible that there are cancer cells with low or no expression of CD44, which will not be detected using our approach. For more comprehensive cancer detection, biomarkers beyond CD44 can be targeted. With the potential for multiplexing, the SERS‐NP strategy can be applied for simultaneous detection of multiple biomarkers in one imaging session, which is a direction we will pursue.

## 3 Conclusions

A facile one‐pot synthesis of SERS‐NPs has been developed, which is applicable to a variety of Raman dyes including both resonant and non‐resonant Raman dyes. Using the seed growth method assisted by tris base, the synthesized 50 nm spherical SERS‐NPs have more homogeneous size and shape distribution, which is a significant improvement compared to those prepared with the more traditional direct sodium citrate assisted synthesis. Bright SERS Raman signals were observed with the NPs synthesized, which could be detected with fM sensitivity. The multiple flavors of the SERS‐NPs available enabled multiplex imaging with the necessary algorithm established to unmix the flavors and accurately determine the ratios of various flavors present.

For biological detection, an important attribute of the SERS‐NPs is their ability to selectively bind with the target of interests. The direct attachment of the targeting ligand HA to SERS‐NPs gave relatively low binding to the target protein CD44. Instead, a new liposomal‐ based synthesis strategy was established to install HA onto the SERS‐NPs, which had significantly higher CD44 binding compared to the corresponding HA‐SERS‐NPs without the liposome formulation. The liposome‐SERS‐HA enabled successful image‐guided removal of breast cancer via surgery from a spontaneous mouse breast cancer model. With their bright Raman signals, availability in multiple flavors, high biocompatibility and colloidal stabilities, and the high targeting abilities, the liposomal SERS particles prepared in this study have powerful translational potential for image guided surgery. Furthermore, the successful application of liposome‐SERS‐HA in real time image‐guided surgery opens a new avenue to investigate polysaccharides as potential targeting agents for SERS applications complementing current antibody‐based strategies.

## 4 Experimental Section

4.1

4.1.1

##### Reagents

Sodium hyaluronan (10 kDa) was purchased from Lifecore Biomedicals. HS‐PEG and HS‐HA of different molecular weight were purchased from Creative PEGWorks. Trisodium citrate dihydrate (C_6_H_5_Na_3_O_7_·2H_2_O, 99.0%), tris base (NH_2_C(CH_2_OH)_3,_ 99.9%), and gold(III) chloride trihydrate (HAuCl_4_·3H_2_O, 99.995%), 4,4′‐dipyridyl (S420), d8‐4,4′‐dipyridyl (S421), trans‐1,2‐bis(4‐pyridyl)‐ethylene (S440), 4‐azobis(pyridine) (S481), 2,5‐bis(4‐pyridyl)‐1,3,4‐thiadiazole (S493), IR780 and Cy5.5, cholesterol as well as other chemicals were purchased from Sigma Aldrich unless otherwise stated. 1,2‐Dipalmitoyl‐sn‐glycero‐3‐PC (DPPC) was obtained from Cayman Chemical. Deionized water (Milli‐Q grade, Millipore) with a resistivity of 18.2 MΩ cm was used throughout the experiment.

##### NP Characterization

The size of the NPs was measured by DLS and surface charge was obtained by ζ potential using a Zetasizer Nano zs apparatus (Malvern, UK). SpectraMax M3 plate reader was used to record UV‐Vis absorption spectra. The TEM images of the NPs were acquired with a TEM (JEM‐ 2200FM) operating at 200 kV using Gatan multiscan CCD camera with Digital Micrograph imaging software.

##### Raman Measurements and Spectral Unmixing Processing

Raman measurement was carried out using both Andor system (Figure S8, Supporting Information) and Renishaw inVia Reflex Raman system. Principal component analysis was employed for spectral unmixing to attain weight values.

##### Sodium Citrate Assisted Synthesis of SERS NPs

A 250 mL Erlenmeyer flask was cleaned extensively by washing with Aqua regia, then with DI water. 100 mL MilliQ water was added to the 250 mL Erlenmeyer flask, boiled and discarded for further washing. Then MilliQ water (48 mL) and 25 mM HAuCl_4_ solution (0.5 mL) were added into the 250 mL Erlenmeyer flask and capped with aluminum foil. For a standard hot plate (Corning PC‐4200), the stirring was set to 500 RPM, and heating was set to 500 °C, for vigorous stirring and heating. Under vigorous stirring, the solution was heated to boiling, followed by the addition of sodium citrate (5 mg mL^−1^, 0.75 mL). Color change occurred within 4 min, indicating the formation of Au‐NPs. Subsequently, a solution of S440, S420, S421, or S481 (5 μL, 1 mM) in DMF was added. Stirring was kept for another 2 min, followed by cooling down in ice bath, and centrifugation at 5000 g at 4 °C for 10 min to collect SERS NP pellet.

##### Tris Base Assisted Synthesis of SERS NPs: Synthesis of 17 nm Seeds

A 250 mL Erlenmeyer flask was cleaned extensively by washing with Aqua regia, then with DI water. MilliQ water (100 mL) was added to the 250 mL Erlenmeyer flask, boiled and discarded for further washing. MilliQ water (48 mL) was added into the Erlenmeyer flask and capped with aluminum foil. For a standard hot plate (Corning PC‐4200), the stirring was set to 500 RPM, and heating was set to 500 °C for vigorous stirring and heating. When the solution started to boil, HAuCl_4_ solution (25 mM, 0.5 mL) was added, followed by the addition of 1% sodium citrate solution (1.5 mL). With the addition of sodium citrate, Au‐NP formation was initiated. Color change occurred within 5 min, indicating the formation of Au‐NPs. The solution was kept stirring for another 8 min after the addition of sodium citrate. Gold colloidal was then cooled down in an ice bath. The volume of the solution was adjusted to 50 mL.

##### Tris Base Assisted Synthesis of SERS NPs: Synthesis of 50 nm SERS NPs

A 250 mL Erlenmeyer flask was cleaned extensively by washing with Aqua regia, then with DI water. MilliQ water (100 mL) was added to the 250 mL Erlenmeyer flask, boiled and discarded for further washing. MilliQ water (91 mL) was added into 200 mL Erlenmeyer flask and capped with aluminum foil. For a standard hot plate (Corning PC‐4200), the stirring was set to 500 RPM, and heating was set to 500 °C for vigorous stirring and rapid heating to achieve boiling temperature within 5 min (the reaction was also performed using a heating mantle or an oil bath with identical results as long as the reaction media reached boiling temperature within 5 min). When the solution started to boil, 0.1 M tris base (4 mL) was added. Then 3 mL seed solution was added, followed by the addition of 25 mM HAuCl_4_ (1 mL). The addition of tetrachloroauric acid initiated the formation of AuNPs. Color change occurred within 3 min. Then 10 μL Raman dye solution (100 mM for S420, S421, S440, S481, and S493, 500 μM for IR780 and Cy5.5) was added and stirring was kept for exactly 1 min. Then gold colloidal was cooled down in ice bath for 3 min, followed by centrifugation at 5000 g at 4 °C for 10 min. Supernatant was carefully removed without disturbing the pellets. SERS NPs were redispersed into MilliQ water. Concentration was measured using calibration curve established with absorption at 527 nm.

##### Competitive ELISA

The competitive ELISA was performed using CD44‐FC *γ* chimera (0.2 μg well^−1^, R&D systems, cat no. 3660‐CD) as the coating antigen following a literature procedure.^[^ ^57^ ^]^ The abilities of HA (10 kDa, 1 μg well^−1^, 100 μL), or liposome‐SERS‐HA (at equivalent HA amount) to compete against b‐HA (0.5 μg well^−1^, 100 μL) for CD44 binding was determined.

##### MTS Assay with SERS‐NPs

4T1 cells (obtained from ATCC, 1 × 10^5^ cells mL^−1^, 200 μL corresponding to 20 000 cells per well) were dispersed in RPMI 1640 cell culture media containing FBS (10%) and cultured in a 96‐well plate for 16 h in the presence of 5% CO_2_ at 37 °C. HC11 cells (obtained from ATCC, 1 × 10^5^ cells mL^−1^, 200 μL corresponding to 20 000 cells per well) were dispersed in RPMI 1640 cell culture media (ATCC modification. Catalog #: A10491‐01) containing FBS (10%) and cultured in a 96‐well plate for 16 h in the presence of 5% CO_2_ at 37 °C. The media was removed, and the cells were washed two times with PBS. The cells were incubated with various concentrations of liposome‐SERS‐PEG or liposome‐SERS‐HA (0, 10, 20, 50, 100, 200, 300, and 500 pM of SERS‐NPs) for 4 h. The SERS‐NP concentration was calculated using the following method: the average number of gold atoms in each particle core is calculated with the formula $\frac{Vn}{M}$, where *V* is volume of the particle (*V* = *π*D^3^/6 where D is the average diameter of the Au core), *ρ* is density of fcc gold (*ρ* = 19.3 g cm^−3^), *n* is the Avogadro number, and *M* is the atomic weight of gold (197 g mol^−1^). The total number of gold atoms in a sample was measured using inductively coupled plasma ‐ optical emission spectroscopy (ICP‐OES). The concentration of the SERS‐NPs was then calculated by dividing the total number of gold atoms in the sample by the average number of gold atoms per particle.

Following incubation of liposome‐SERS‐PEG or liposome‐SERS‐HA, the supernatants were removed, and cells were washed with PBS two times. MTS reagent (Promega, cat no. G358C) dispersed in medium (17%) was added and incubated for another 3 h in the dark. A brown color appeared in the wells containing live cells. The absorption of each well was measured at 490 nm using a SpectraMax M3 plate reader, with the absorbance from wells without cells (blanks) subtracted as background from each sample.

##### Synthesis of Liposome‐SERS‐HA/PEG

Liposome‐SERS complexes were prepared through a modified thin film hydration method. Briefly, DPPC (2 mg) and cholesterol (0.5 mg) were dissolved in CHCl_3_/MeOH (v/v: 2/1; 0.5 mL), and dried using a rotary evaporator at 37 °C to form a lipid film. The film was hydrated with SERS‐HA or SERS‐PEG solution. For SERS‐HA, SERS NP (200 pM, 500 μL) was mixed with 100 μL, 10 mg mL^−1^ HS‐HA (10 kDa) and incubated overnight. For SERS‐PEG, SERS NPs (200 pM, 500 μL) were mixed with HS‐PEG (5 kDa, 10 μL, 10 mg mL^−1^) and incubated overnight. During hydration, the solution was kept still for 30 min without sonication. Then the solution was pipetted up and down to facilitate the hydration process followed by water bath sonication. The complex was centrifuged down at 2500 g for 5 min, washed two times by repeated centrifugation to remove unbounded PEG, HA, and empty liposome, and redispersed back into MilliQ water.

##### Stability of Liposome‐SERS‐HA/PEG in PBS

Two solutions of liposome‐SERS‐HA/PEG (1 mL, 200 pM) dissolved in water were placed in glass vials. A phosphate‐buffered saline solution (0.1 mL) was added to the vials with final PBS concentrations of 1x (8 mM NaH_2_PO_4_, 150 mM NaCl, 3 mM KCl, and 2 mM KH_2_PO_4_ at pH 7.4) and 10× PBS respectively. UV‐Vis and DLS measurements were carried out before and after the addition of PBS solution.

##### Stability of Liposome‐SERS‐HA/PEG in Serum

An aqueous solution of liposome‐SERS‐HA/PEG in water was introduced to undiluted mouse serum in a 1:1 v/v ratio, followed by a 24‐hour incubation at 37 °C. Afterward, the samples were centrifuged at 5000 g, and the supernatants were removed. The samples were then re‐suspended in distilled water and centrifuged again at 5000 g. The precipitates were collected and re‐suspended, and UV‐Vis and DLS measurements were conducted.

##### Carbazole Assay for Hyaluronan Quantification

The sample (50 μL) was mixed with sodium tetraborate in sulfuric acid (25 mM, 200 μL) in a 0.7 mL tube, and heated at 100 °C for 10 min. This was followed by the addition of 0.125% carbazole in absolute ethanol (50 μL) after the sulfuric acid solution cooled down. The mixture was then heated at 100 °C for another 10 min. After cooling down to room temperature, the mixture (200 μL) was transferred into wells in a 96 well microtiter plate, and the optical densities at 550 nm were measured.

##### Cell Culture for Raman Imaging

Breast cancer cell line, 4T1cells (≈0.2 × 10^6^ cells mL^−1^) were grown on custom‐made (6 mm × 6 mm × 1 mm thick, CHEMGLASS Microscope Slide, GRAINGER) quartz‐bottomed 100 mm Petri dish containing 13 mL RPMI 1640 culture medium supplemented with 10% v/v FBS and 1% penicillin‐streptomycin in an incubator at 37 °C with 5% CO_2_ for 2 days. Cells grown on quartz were transferred into 48 well‐plate and fixed with 10% neutral buffered formalin solution upon the incubation for 30 min at 4 °C following washing with PBS two times. The cells were incubated with liposome‐SERS‐HA/PEG (100 pM) for 3 h at 4 °C with a gentle shaking of 90 rpm following two times washing by PBS.

##### Image‐Guided Surgery Through SERS Imaging

The staining solution was prepared by mixing Liposome‐S421 SERS‐HA (500 pM) and Liposome‐S440 SERS‐PEG (500 pM) at 1:1 ratio. MMTV‐PyMT transgenic mice were purchased from the Jackson Laboratory. In a span of 4 months, the female mice showed the spontaneous onset of palpable breast cancer. The mice were housed at Michigan State University's Laboratory Animal Resources Facility. All activities and protocols related to the animal study received approval from the Institutional Animal Care and Used Committee (IACUC) at Michigan State University. An MMTV mouse (body weight ≈28 g; tumor size: 100–200 mm^2^; the weight of the tumor was below 10% of the body weight of the mice following the IACUC guidelines) was put under anesthesia with 5% isoflurane in oxygen. The tumor area was first opened, and the tissue background was scanned. Then 50 μL of the staining solution was topically applied to both the tumor area and surrounding normal area. After 15 min, the staining area was washed with PBS three times. Raman mapping of the tissue area was obtained before and after the washing process. Then a portion of the tumor was removed, followed by repeated staining and imaging. The whole process is repeated until all the tumor is removed. Dissected tissue was fixed with paraformaldehyde (4%) for H&E and IHC staining. The experiments were performed on three mice with similar results, and the representative images were shown in Figure 5. All animal experiments were performed in accordance with the guidelines of the IACUC of Michigan State University (approved protocol #: PROTO202100095).

##### Statistical Analysis

All data were represented as mean ± standard deviation (SD) (*n* = 3) unless specified otherwise. Statistical significance between two groups was assessed using two‐way ANOVA and the significance level was set at a value of 0.05. The data were indicated as ns: *P* > 0.05; *: *P* < 0.05. The statistical analysis was performed using GraphPad.

## Conflict of Interest

The authors declare no conflict of interest.

## Supporting information

Supplementary Material

## Data Availability

The data that support the findings of this study are available in the supplementary material of this article.
