# Supplementary material for: Robust Synthesis of Targeting Glyco‐Nanoparticles for Surface Enhanced Resonance Raman Based Image‐Guided Tumor Surgery
Source: Small Sci. 2024 Feb 22;4(5):2300154. doi: 10.1002/smsc.202300154 (PMC11340905; doi:10.1002/smsc.202300154)
Supplement: Supplementary file 1 — Supplementary Material [file SMSC-4-2300154-s001.pdf]

Supporting Information

Robust Synthesis of Targeting Glyco-nanoparticles for Surface Enhanced Resonance Raman Based Image-Guided Tumor Surgery

Kunli Liu,[a,b,#] A. K. M. Atique Ullah,[a,b,#] Aniwat Juhong,[b,c] Chia-Wei Yang,[a,b] Cheng-You Yao,[b,c] Xiaoyan Li,[d] Harvey L. Bumpers,[e] Zhen Qiu,[b,c,f]* Xuefei Huang[a,b,f]*

[a]Department of Chemistry, Michigan State University, East Lansing, MI, 48824 USA
[b]Institute for Quantitative Health Science and Engineering, Michigan State University, East Lansing, MI, 48824 USA
[c]Department of Electrical and Computer Engineering, Michigan State University, East Lansing, MI, 48824 USA
[d]Department of Civil and Environmental Engineeing, Michigan State University, East Lansing, MI, 48824 USA
[e]Department of Surgery, Michigan State University, East Lansing, MI, 48824 USA
[f]Department of Biomedical Engineering, Michigan State University, East Lansing, MI, 48824 USA
[#]Equal contribution
Email: qiuzhen@msu.edu; huangxu2@msu.edu

Table of Contents

**Figure S1**.	S3
**Figure S2**.	S4
**Table S1**.	S5
**Figure S3**.	S6
**Figure S4**.	S7
**Figure S5**.	S8
**Figure S6**.	S9
**Figure S7**.	S10
**Figure S8**.	S11
**Figure S9.**	S12

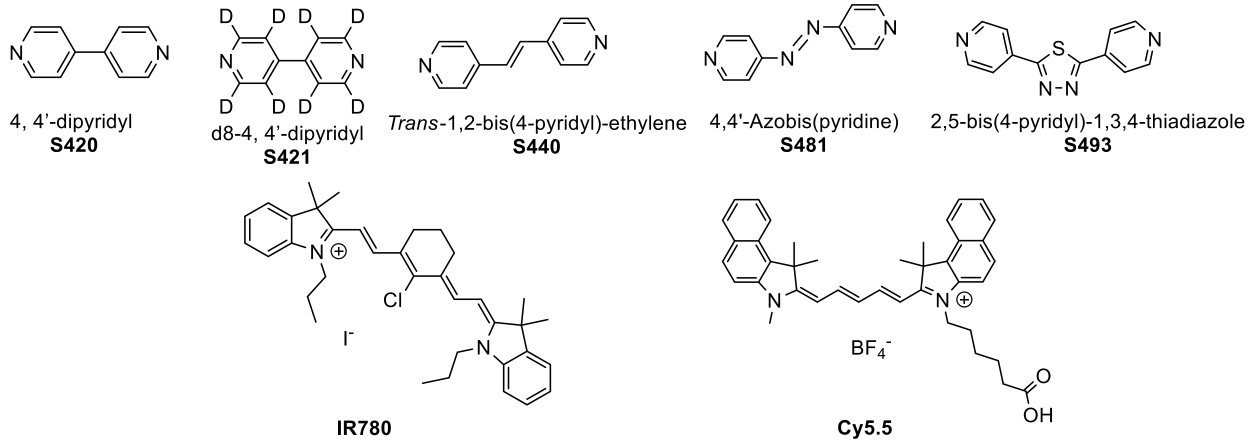

Figure S1. Structures of non-resonant dyes (S420, S421, S440, S481, and S493) and resonant dyes (IR780, and Cy5.5) examined for SERS-NP formation.

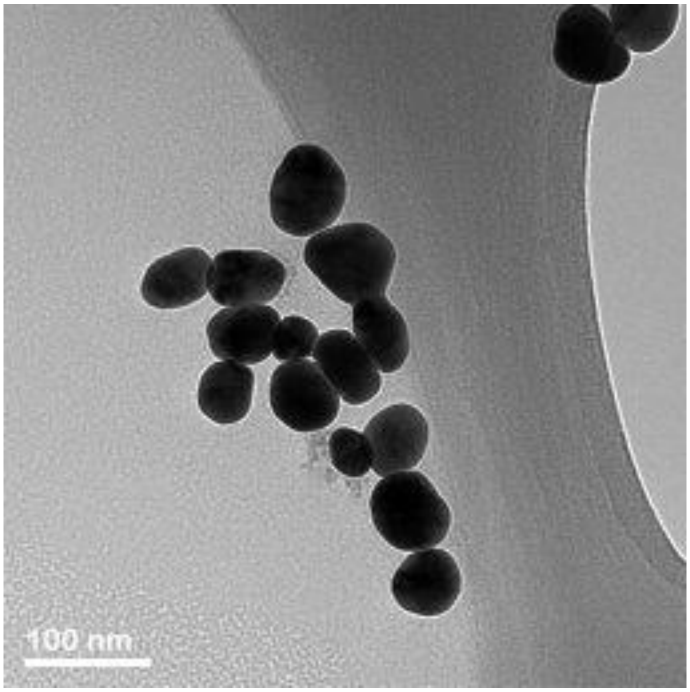

Figure S2. Representative TEM image of sodium citrate reduction mediated Au NPs. Significant heterogeneities in shape and size were observed with NPs synthesized via this method.

Table S1. ζ potential measurements (mean ± standard deviation, N =3).

Samples	Zeta Potential (mV)
Au NP Seed	-41 ± 2
S420 SERS-NPs	-36 ± 2
S481 SERS-NPs	-35 ± 3
PEG-S420 SERS-NPs	-22 ± 1
HA-S481 SERS-NPs	-28 ± 2
Liposome-S420 SERS-PEG	-13 ± 4
Liposome-S481 SERS-HA	-24 ± 3

(a)

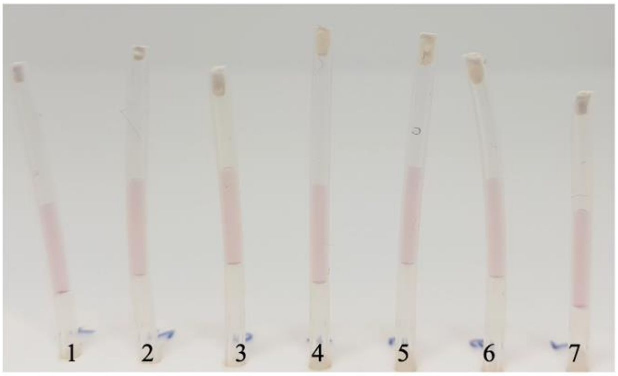

(b)

Tube No	Ground truth Ratio (S420 : S481 : S421)	Computed Ratio (S420 : S481 : S421)
1	1.0 : 1.0 : 1.0	1.0 : 1.0 : 1.2
2	1.0 : 1.0 : 0.0	1.0 : 1.1 : 0.0
3	1.0 : 0.0 : 1.0	1.0 : 0.0 : 1.2
4	2.0 : 1.0 : 1.0	2.0 : 1.0 : 1.1
5	2.0 : 1.0 : 0.0	1.6 : 1.0 : 0.0
6	2.0 : 0.0 : 1.0	1.8 : 0.0 : 1.0
7	1.0 : 2.0 : 1.0	1.0 : 2.2 : 1.0

Figure S3. The ratios of SERS-NPs in the mixed NP solutions can be accurately determined via a custom-designed unmixing algorithm. (a) Photo of the phantoms with various mixed SERS-NPs (S420, S481, and S421) solutions; and (b) the result of NP unmixing applying the demultiplex algorithm.

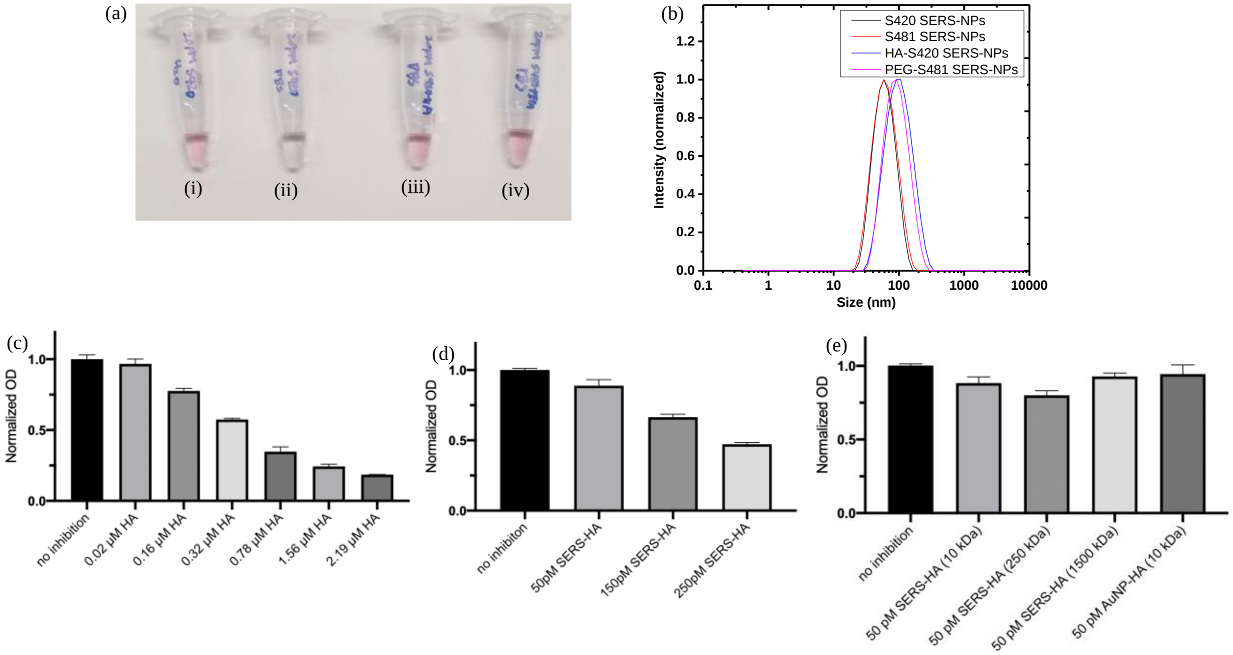

Figure S4. Characterizations of HA-SERS-NPs and PEG-SERS-NPs. a) Photos of SERS-NPs, HA-SERS-NPs and PEG-SERS-NPs in MilliQ water or PBS: (i) 20 pM S420 SERS-NPs in MilliQ water, (ii) 20 pM S420 SERS-NPs in PBS. Aggregation is indicated by the color change from red to black and precipitation of the aggregate at the bottom of the Eppendorf tube. (iii) 20 pM HA-S420 SERS-NPs in PBS and (iv) 20 pM PEG-S481 SERS-NPs in PBS. HA or PEG attachment to SERS-NPs significantly improved colloidal stability of SERS-NPs in PBS. (b) DLS data of SERS-NPs before and after ligand HA/PEG attachment. After the ligand attachment, the size of the SERS-NPs was increased with uniform size distribution. (c) HA competition with b-HA at various concentrations; (d) Competition of HA-SERS-NPs (10 kDa) were observed against b-HA for CD44 binding. HA-SERS-NPs showed weak competition even at elevated concentration at 250 pM. (e) Competition of 50 pM HA-SERS-NPs with HA at different molecular weights: 10 kDa, 250 kDa and 1500 kDa. Similar results were observed across 3 different molecular weights.

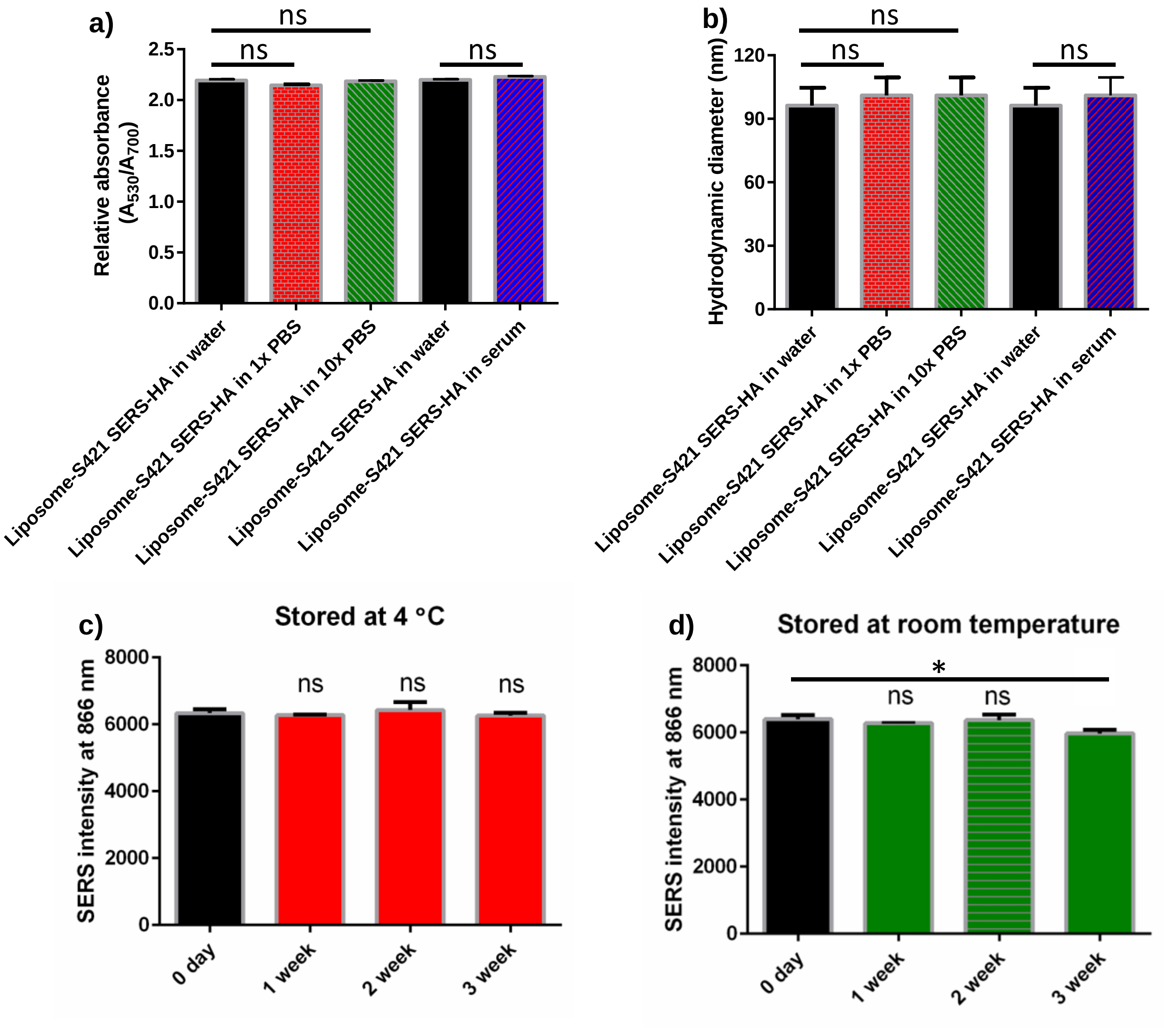

Figure S5. Analysis of particle stability. a) Relative absorbances and b) hydrodynamic diameters of the liposome-S421 SERS-HA particles in water, 1x PBS, 10x PBS, and serum. No statistically significant changes in the relative absorbance data or the hydrodynamic diameters suggested the stability of the NPs in PBS and serum. SERS signal intensities of liposome-S421 SERS-HA stored at c) 4 °C or d) room temperature over 3 weeks. For each column, the mean value with the standard deviation was plotted. For each concentration, the values were from three samples. No statistically significant change in SERS intensity was observed for the NPs stored at 4°C up to 3 weeks. NPs stored at room temperature showed some changes in SERS intensities only at the 3-week mark. Statistical analysis was performed using a two-way ANOVA. ns: $P > 0.05$. *: $P < 0.05$.

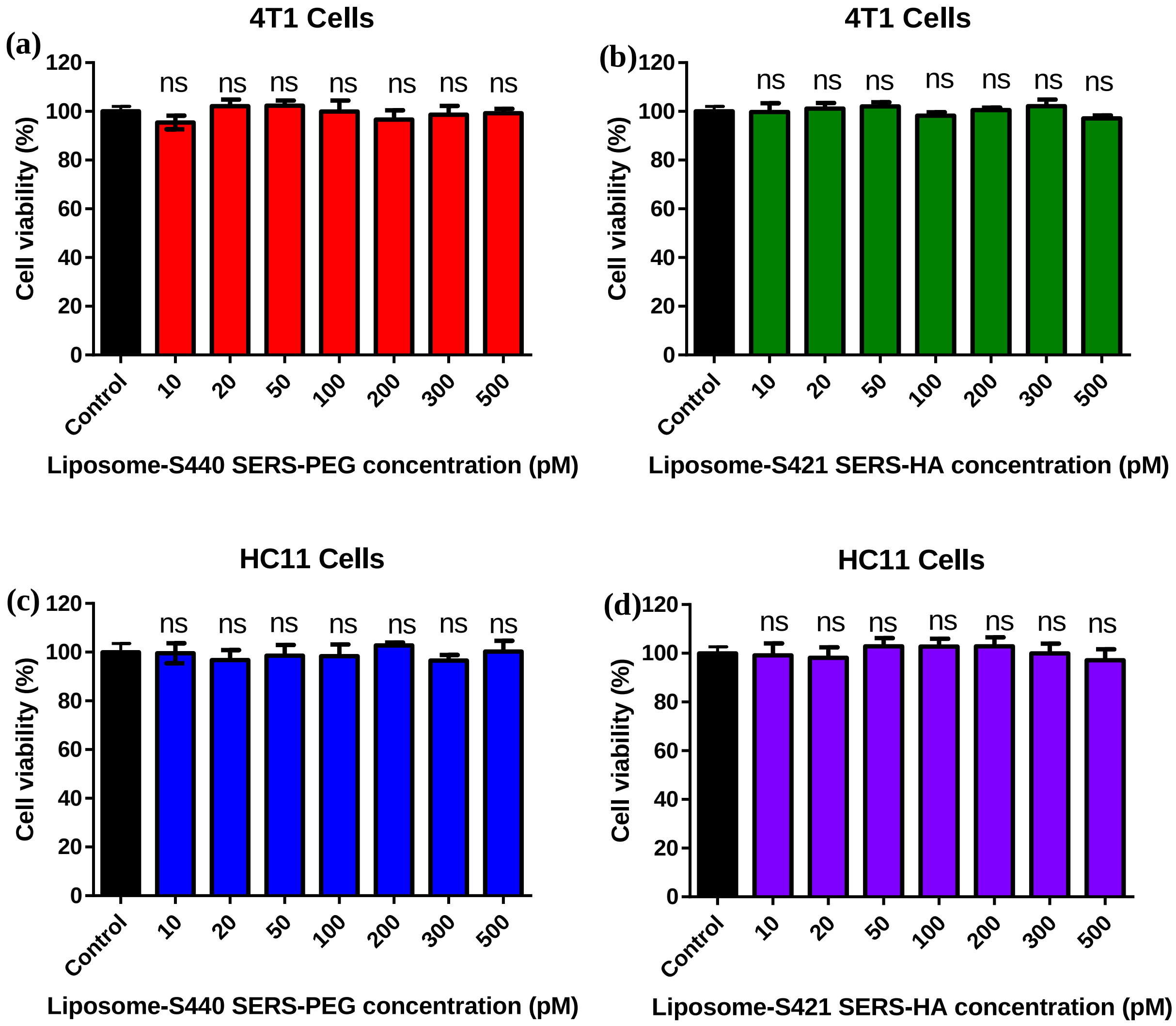

Figure S6. MTS assays with 4T1 breast cancer cells and HC11 mouse normal mammary epithelial cells indicated the NPs did not affect cell viabilities under the experimental condition. 4T1 cells were incubated with a) liposome-S440 SERS-PEG or b) Liposome-S421 SERS-HA; and HC11 cells were incubated with (c) liposome-S440 SERS-PEG and (d) liposome-S421 SERS-HA for 4 h under various NP concentrations: 0 pM, 10 pM, 20 pM, 50 pM, 100 pM, 200 pM, 300 pM, and 500 pM. No significant changes in cell viability were observed suggesting the NPs were not toxic to breast cancer cells or normal mammary cells up to at least 500 pM. For each column, the mean value with the standard deviation was plotted. For each concentration, the values were from three samples. Statistical analysis was performed using a two-way ANOVA. ns: P > 0.05.

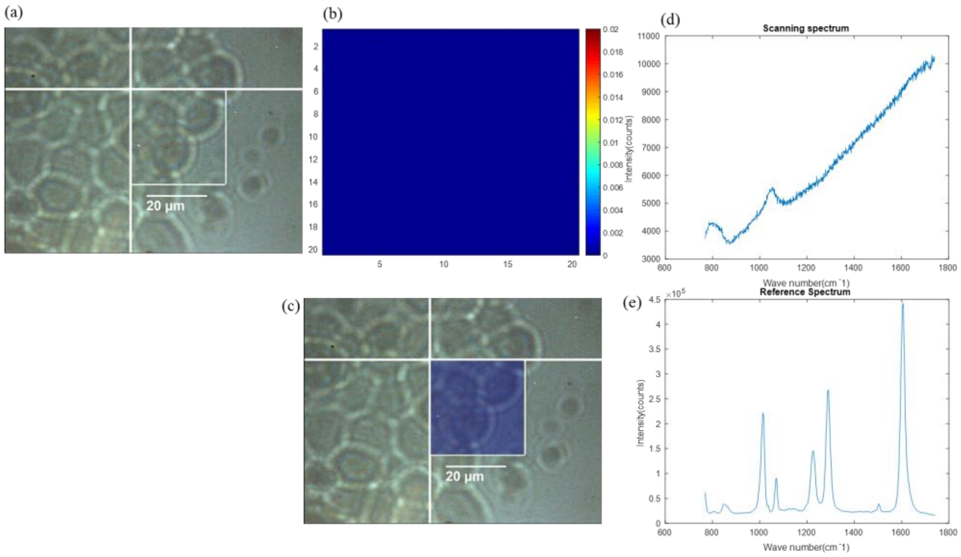

Figure S7. Images of 4T1 cells incubated with liposome-S420 SERS-PEG followed by washing to remove unbound particles. (a) Bright-field microscopy image of 4T1 cells, (b) S420 SERS image of 4T1 cells, (c) overlay image of 4T1 cells, (d) mean SERS spectrum of several spectra at different points scanned from the cells area and empty area, and (e) reference spectrum of S420 SERS Au NPs. The cells were incubated with liposome-S420 SERS-PEG, considering a control experiment (without targeting) and the spectra were recorded using 40X water immersion lens. The recorded number of spectra were 400 with 20 x 20 number of pixels, 5 frames accumulation, 2 µm step size, and 1 s exposure time. The colorimetric weight factor of the SERS image (b) and mean SERS spectra (c), indicate that there was no SERS signal obtained from the cell area as well as the empty area demonstrating the non-specific binding of liposome-SERS-PEG with the cells.

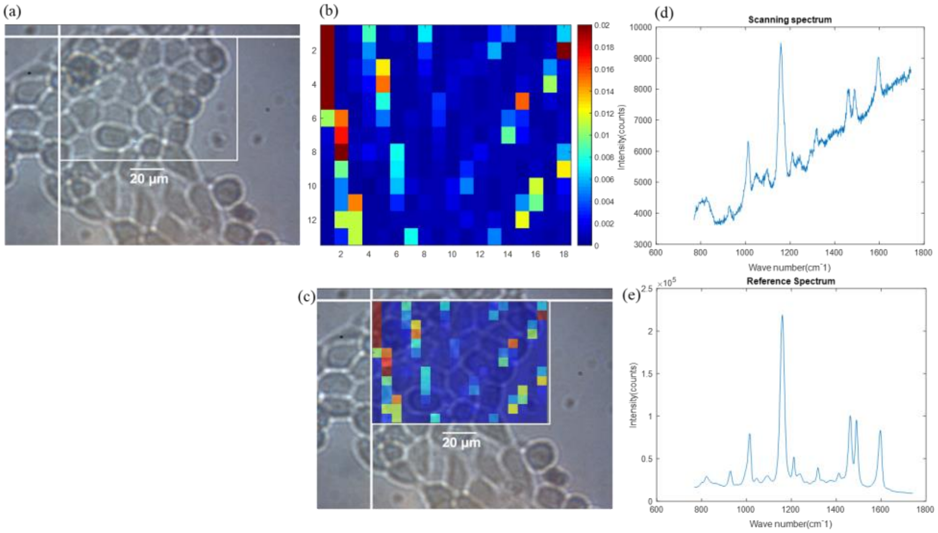

Figure S8. Images of 4T1 cells incubated with liposome-S481 SERS-HA followed by washing to remove unbound particles. (a) Bright-field microscopy image of 4T1 cells, (b) S481 SERS image of 4T1 cells, (c) overlay of images of SERS with 4T1 cells, (d) Average SERS spectrum of several spectra at different points in the cells area and empty area, and (e) Reference spectrum of S481 SERS-NPs. The cells were incubated with liposome-S481 SERS-HA considering a targeting of CD44 protein, overexpressed in breast cancer cells and the spectra were recorded using 40X water immersion lens. The numbers of spectra were 234 with 18 x 13 number of pixels, 5 frames accumulation, 5 µm step size, and 1 s exposure time. The colorimetric weight factor of the SERS image (b) and mean SERS spectra indicated that the SERS signals were obtained only from the cell area indicating the targeting of HA to 4T1 cells.

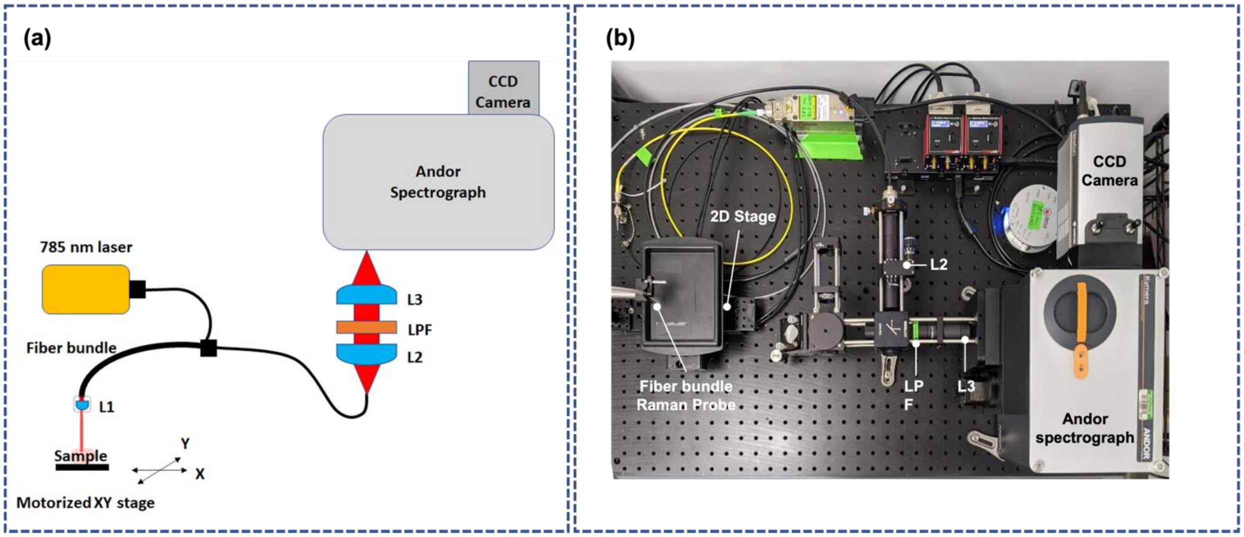

Figure S9 (a) Schematic configuration of the fiber bundle Raman spectrometer. (b) Photograph of the fiber bundle Raman spectrometer. A 785 nm laser (iBeam Smart 785, Toptica Photonics, Munich, Germany) is applied to illuminate the sample. The scattered Raman spectra is acquired by the custom-made fiber bundle Raman probe (Fiber guide Industries, Caldwell, ID, USA). The lens L1 (f=6.83 mm, PLCS-4.0-3.1-UV, CVI Laser Optics, Albuquerque, NM, USA) is used to collimate both excitation (785 nm laser) and emission (scattered Raman spectra) through the fiber bundle Raman probe. An optical relay, containing two lenses (L2, L3) and a long pass filter (LPF), couples the light into the slit of the spectrometer (Kymera 193i-A, Andor Technology, Belfast, UK) and CCD camera (1024 pixel x 256 pixel with pixel size of 26 µm x 26 µm; DU920P Bx-DD, Andor technology, Belfast, UK).
